# TRIM28 Regulates Proliferation of Gastric Cancer Cells Partly Through SRF/IDO1 Axis

**DOI:** 10.7150/jca.95094

**Published:** 2024-06-11

**Authors:** Zhiye Huang, Jiaxing Dong, Taohua Guo, Wanju Jiang, Renhao Hu, Shun Zhang, Tao Du, Xiaohua Jiang

**Affiliations:** 1 School of Medicine, Tongji University, Shanghai, 200092, China.; 2 Department of Gastrointestinal surgery, East Hospital, Tongji University School of Medicine, Shanghai, 200120, China.

**Keywords:** gastric cancer (GC), TRIM28, IDO1, SRF, cell proliferation

## Abstract

**Background:** Gastric cancer (GC) is one of the most common malignancies worldwide, with high incidence and mortality rate. Tripartite motif-containing 28 (TRIM28) is an important molecule that affects the occurrence and development of tumors, but its function in GC has not been elucidated clearly. The purpose of this study is to explore the molecular mechanism by which TRIM28 affect the GC.

**Methods:** TRIM28 expression was tested in RNA-seq data from TCGA database, tumor tissue samples from patients and GC cell lines. Genes were silenced or overexpressed by siRNA, lentivirus-mediated shRNA, or plasmids. Cell Counting Kit-8 (CCK-8) and colony formation assays were performed to explore the proliferation of GC cells after TRIM28 knockdown. RNA-seq and TCGA database were used to identify target genes. Luciferase report assay was employed to detect the possible mechanism between TRIM28 and Indoleamine 2,3-dioxygenase (IDO1). Tryptophan concentration in cell supernatant was measured using a fluorometric assay kit. MGC-803 and 746T cells were injected into mice to establish xenograft animal models.

**Results:** The expression of TRIM28 was positively correlated with tumor size and poorer prognosis. Upregulation of TRIM28 was observed in GC tissues and cells. *In vitro*, we proved that knockdown of TRIM28 significantly inhibited the proliferation of GC cells. Then TRIM28 was found to be positively correlated with the expression of IDO1 in GC cells. In accordance with this, tryptophan levels in cell supernatants were increased in TRIM28 knockdown GC cells and overexpression of IDO1 could reverse this phenotype. Serum response factor (SRF), a reported regulator of IDO1, was also regulated by TRIM28 in GC cells. And decreased expression of IDO1 induced by TRIM28 knockdown could be partly reversed through overexpression of serum response factor (SRF) in GC cells. Functional research demonstrated that the expression of IDO1 was increased in GC and IDO1 knockdown could also inhibited the proliferation of GC cells. Furthermore, overexpression of IDO1 could partly reverse proliferation inhibited by TRIM28 knockdown in GC cells. *In vivo*, knockdown of TRIM28 significantly inhibited the tumor growth and overexpression of IDO1 and SRF both could reverse proliferation inhibited by TRIM28 knockdown.

**Conclusions:** TRIM28 is crucial in the development of GC, and may regulate IDO1 through SRF. TRIM28 promote GC cell proliferation through SRF/IDO1 axis.

## 1. Background

Gastric cancer (GC) ranks among the prevalent malignant tumors globally, characterized by elevated mortality and incidence rates 1. The methods that are used to treat GC include surgery, chemotherapy, and molecular targeted therapy, but postoperative recurrence and metastasis are the main causes of treatment failure 2. On the other hand, due to the asymptomatic characteristics of the early stages of GC, diagnosis is often delayed, which further reduces the five-year survival rate 3. Therefore, exploring novel therapeutic targets of GC to improve the clinical diagnosis and treatment of GC has important clinical significance.

The tripartite motif (TRIM) family is a family of proteins with a conserved structure, and 65 family members have been identified in the human genome 4. Tripartite motif-containing 28 (TRIM28), is an important member of the TRIM family and can regulate various biological processes, such as development, cell proliferation, aging, DNA repair and cell differentiation 5-7. Studies have shown that TRIM28 is differentially expressed in several cancers, such as lung cancer, liver cancer, prostate cancer, GC and colorectal cancer, and affects biological processes such as tumor cell proliferation, differentiation, apoptosis, invasion and migration 8-10. It was reported that TRIM28 could promote the escape of GC cells from immune surveillance 11, suppress cancer stem-like characteristics 12 and is associated with peritoneal carcinomatosis in GC cells 13. However, the molecular mechanisms of TRIM28 on GC development deserve further investigation.

Indoleamine 2,3-dioxygenase (IDO1), which is also known as indoleamine 2,3-dioxygenase 1, is one of the key enzymes that regulates tryptophan metabolism in the body 14. IDO1 can regulate the immune response by consuming tryptophan in inflammatory environments and the tumor microenvironment. IDO1 plays a role in suppressing effector T and NK cells while also influencing the activation of regulatory T (Treg) cells and myeloid-derived suppressor cells (MDSCs) 15-17, and it is considered as an immune checkpoint. Tumor cells can also utilize the function of IDO1 to suppress the immune response of the body. On the other hand, IDO1 can increase the expression of IL-6 and activate the STAT3 pathway in tissues by consuming tryptophan to produce canine uric acid, thereby upregulating the expression of the downstream gene VEGF and promoting tumor angiogenesis 18. An increasing number of studies have validated its regulatory role in tumors proliferation 19. Such as it was reported that IDO1 metabolites activate β-catenin signaling to promote cancer cell proliferation and colon tumorigenesis in mice 20. Astragaloside IV Inhibits the proliferation of human uterine leiomyomas by targeting IDO1 21. Great potential is displayed by IDO1 in tumor proliferation regulation, which may become novel therapy targets in tumor treatment 22,23.

In this study, we confirmed that TRIM28 and IDO1 are abnormally overexpressed and that their expression levels are positively correlated in GC. Serum response factor (SRF) may mediate the regulation of TRIM28 on IDO1 expression. And TRIM28 may exert its regulative effect on proliferation of GC cells partly through IDO1.

## 2. Materials and methods

### 2.1. Expression of TRIM28 and IDO1 in TCGA-STAD

FPKM matrix of TCGA gastric adenocarcinoma was downloaded from XENA website (https://xenabrowser.net/datapages/). Subsequently, the TPM expression matrix was extracted and ggplot2 package (version:3.3.6) was performed to visualize the mRNA expression of TRIM28 and IDO1 in GC.

### 2.2. Analysis of survival

Kaplan-Meier plotter 24 was used to plot survival curves of TRIM28 in GC, including 875 patients. When the *P*-value < 0.05, it is considered to be significantly correlated with the prognosis of GC.

### 2.3. Ethical statement

This study was approved by the ethics committee of Shanghai East Hospital (Permit number: 2023-113). All animal experiments were approved by the Experimental Animal Ethics Committee of Shanghai East Hospital and performed according to the Guide for the Institutional Animal Care and Use Committee of Shanghai East Hospital (Shanghai, China) (Permit number: 2023-114).

### 2.4. Clinical samples

The specimens of GC patients were collected from Shanghai East hospital from January 2018 to December 2021. No patients received chemotherapy or radiotherapy before the operation, and all were confirmed to have GC through postoperative pathology. Informed consent was obtained from all patients and the study complied with the standards of the Ethics Committee.

### 2.5. Cell culture and reagents

GC cell lines MGC-803, Hs-746T, AGS, HGC-27, and GES-1 preserved in the laboratory, and cultured in RPMI-1640 medium (GIBCO, USA) containing 100u / ml penicillin, 100ug / ml streptomycin and 10% fetal bovine serum (FBS) at 37°C in air with 5% CO_2_ saturation. IFN-γ was purchased from R&D system (MN, USA).

### 2.6. Plasmids and transfection

LV-sh-TRIM28 lentivirus was from Shanghai Genepharma (Shanghai, China). C-tagged Flag TRIM28 plasmid, C-tagged Flag IDO1 plasmid, human SRF Lentivirus (EX-F0287-Lv105-Untagged) and IDO1 promoter plasmid (Gaussia luciferase, HPRM44127-PG04) was purchased from Guangzhou iGeneBio (Guangzhou, China). The plasmid was transfected using lipofectamine 3000 (Invitrogen, MD, USA). The target sequence of TRIM28-siRNA was 5'-UGGCUCUGUUCUCUGUCCUTT-3', and the IDO1-siRNA sequence was 5'-GCGUCUUUCAGUGCUUUGATT-3', the SRF-siRNA sequence was 5'-GCAAGGCACUGAUUCAGACTT-3', the negative control sequence was 5'-UUCUCCGAACGUGUCACGUTT-3', and siRNA was transfected using Lipofectamine RNAi MAX (Invitrogen, MD, USA).

### 2.7. Immunohistochemical (IHC) staining

Utilizing a highly sensitive immunohistochemical approach with streptavidin-biotin-peroxidase, we examined the tissue microarray of GC. Anti TRIM28 (Santacruz, TX, USA) polyclonal antibody was diluted with 1:100. H-score was performed by Quant center software as previous 25. DAB system was applied to stain tissue specimens. Image J (https://imagej.net/ij/) was used to determine the average optical density (AOD).

### 2.8. Reverse transcription and quantitative (RT-qPCR)

RNA was extracted with RNAiso (TaKaRa, Shiga, Japan) and cDNA was synthesized with PrimeScript RT reagent Kit with gDNA Eraser (TaKaRa, Shiga, Japan) according to the instructions. Q-PCR was performed using SYBR Premix EX TaqTM (TaKaRa, Shiga, Japan) and detected by Applied Biosystems 7500 fast sequence detection system (ABI, SD, USA). 18S RNA was used as internal reference and the primers of relative genes were presented in **Table A1.**

### 2.9. Western blotting analysis

Cell lysis was performed using the highly efficient RIPA buffer (Pierce, IL, USA) supplemented with protease and phosphatase inhibitors (CST, MA, USA). Following electrophoresis, proteins were transferred onto a PVDF membrane and subsequently blocked using TBST buffer containing 5% non-fat dry milk and incubated overnight with primary antibody. Protein was incubated with HRP-labeled secondary antibody for 1h and visualization was achieved with ECL chemiluminescence (BioRad, CA, USA). TRIM28 antibody (sc-33186), Flag antibody (sc-166355) was obtained from Santa Cruz (TX, USA). IDO1 antibody (#86630), SRF antibody (#5147), GAPDH antibody, HRP-labeled rabbit and mouse secondary antibodies were from Cell Signaling Technology (MA, USA).

### 2.10. CCK-8 assay and colony formation

Following treatment, CCK-8 reagent was introduced into a 96-well plate containing GC cells, and OD450 was measured after a 2-hour incubation.

For colony formation assay, 1000 cells were seed into 10cm dish and cultured for 2 weeks. Cell clones were fixed with 4% paraformaldehyde, stained with crystal violet, counted and photographed under microscope.

### 2.11. Transcription reporter assay

746T cells were initially plated into 24-well plates, followed by transfection with IDO1 promoter plasmids and TRIM28 siRNA for a duration of 48 hours. Subsequently, the cell supernatants were harvested for the assessment of luciferase activity using Secrete-Pair™Dual Luminescence Assay Kit (GeneCopoeia, Guangzhou, China). Gaussia Luciferase (GLuc) and Secreted Alkaline Phosphatase (SEAP) activities were measured by Substrate Gluc and Substrate SEAP, respectively. The GLuc/ SEAP ratio is the relative activity value of the promoter.

### 2.12. Measurement of Tryptophan concentrations

Tryptophan (Trp) concentration in cell supernatant was determined by Tryptophan Fluorometric Assay Kit (ab211098) (Abcam, MA, USA). Briefly, the cell culture medium was centrifugated at 3000 rpm for 5 min at 4°C, and the supernatant was collected. Standard curves were prepared using the standards in the kit. The samples were incubated with Trp catalyst at 105°C for 60 min and then incubated on ice for 10min according to the instructions. And the fluorescence value of 370/440 was measured and the tryptophan concentration was obtained by referring to the standard curve.

### 2.13. *In vivo* tumorigenesis

Male BALB/C nude mice were accommodated in a Specific pathogen free (SPF) environment at the Animal Center. The research adhered to the guidelines of experimental animal law. Subcutaneous injection of 1×10^6^ cells was performed in 4-week-old male nude mice, with seven mice in each group. Tumor diameter was monitored at 7-day intervals, and tumor volume was computed using the formula: volume = (W^2 × L)/2. Mice were sacrificed via isoflurane induction after 30 days following the approved IACUC protocol.

### 2.14. Statistical analysis

The data were shown as mean ± SE and were processed by GraphPad Prism 9 software (GraphPad InStat Software, CA, USA), R software (version:4.3.0; https://cran.r-project.org/) and SPSS software (version:23.0). The expression of TRIM28 and clinical parameters were analyzed by Pearsonχ2 test. One-way ANOVA and Tukey's multiple comparison test was used to compare the experimental groups. Kaplan-Meier survival curves and log-rank tests were conducted to explore the correlation between TRIM28 and overall survival rate (OS). The statistically significant of two-tailed *P* value were set at *P < 0.05 and **P < 0.01.

## 3. Results

### 3.1. TRIM28 expression is increased in GC tissues and cells

**Figure 1:**
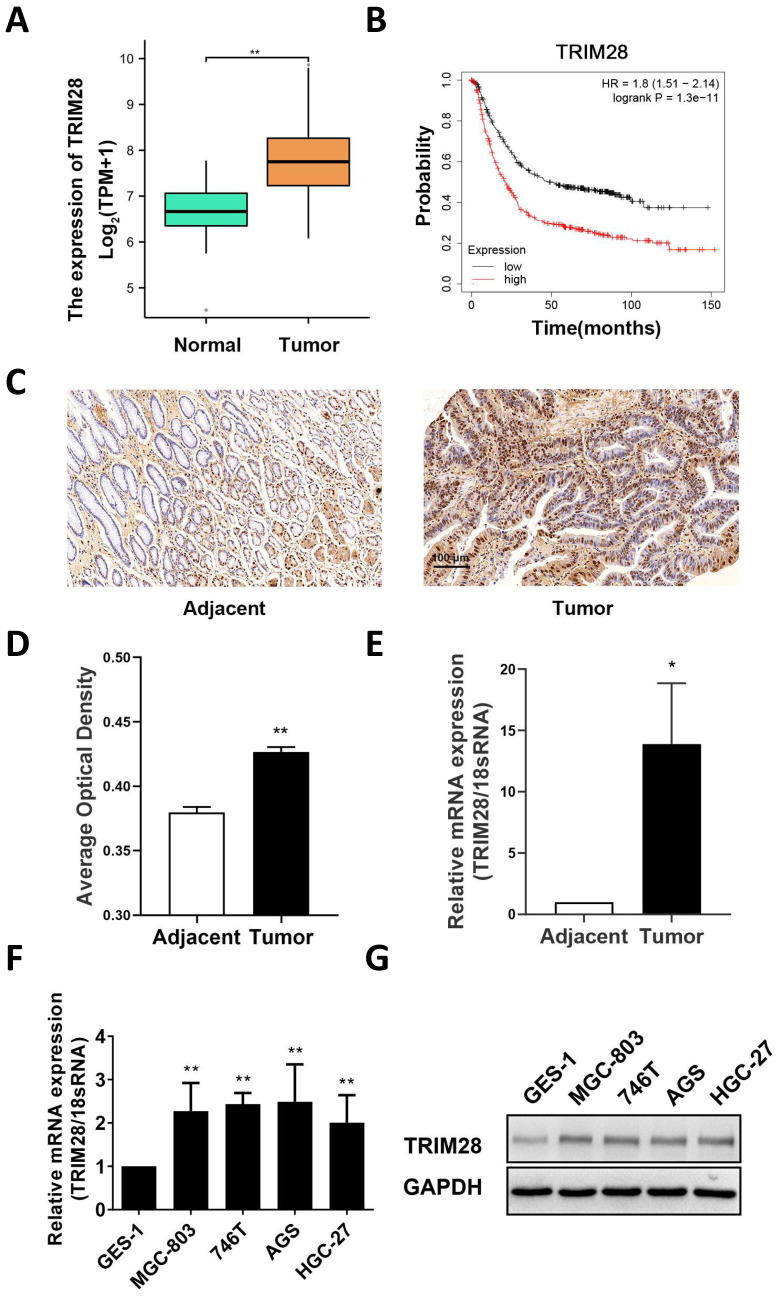
** TRIM28 expression is increased in GC tissues and cells.** (A) Comparison of TRIM28 mRNA expression between GC and adjacent tissues in the TCGA database. (B) Line plot showing the Kaplan‒Meier analysis results of the survival rate of patients in the high TRIM28 expression and low TRIM28 expression groups. (C) Immunohistochemical staining of TRIM28 in GC and adjacent tissues. Magnification: 200×; Scale bar: 100 μm). (D) Histogram plot showing the average optical density of immunohistochemical staining in different groups (n=76). (E) mRNA expression of TRIM28 in GC and adjacent tissues was measured by qPCR. (F) Histogram plot showing the mRNA expression of TRIM28 in GC cell lines was measured by qPCR. (G) The protein expression of TRIM28 in GC cell lines was measured by WB. All the experimental results are shown as the mean ± SE of at least three independent biological replicates (n = 3) for each group. *P < 0.05; **P < 0.01.

First, a bioinformatics approach was used to analyze the expression of TRIM28 in TCGA database, and the results revealed that the mRNA expression of TRIM28 in GC was significantly higher than that in adjacent tissues (*P* < 0.01, Fig. 1A). Survival analysis of GC patients was conducted with the Kaplan‒Meier Plotter Database, and the results showed that high TRIM28 expression was associated with a poorer prognosis (HR = 1.8, *P* < 0.05, Fig. 1B). The protein expression of TRIM28 in 76 pairs of GC tissues and adjacent tissues was measured by immunohistochemistry. The results showed that the protein expression of TRIM28 was upregulated in GC tissue, and the difference was significant (*P* < 0.01, Fig. 1C-D). Subsequently, qRT‒PCR was used to measure TRIM28 expression in patient tissues, and the results showed that TRIM28 mRNA expression was also upregulated in GC (*P* < 0.05, Fig. 1E), which is consistent with previous results from the TCGA database. Furthermore, we divided GC tissues into a TRIM28-positive group (n = 53) and a TRIM28-negative group (n = 23). Based on the clinical and pathological characteristics of the patients, the results suggested that TRIM28 protein expression was related to tumor size (*P* < 0.05, Table 1). Moreover, these results were validated in GC cell lines, and the results showed that the mRNA and protein expression of TRIM28 was highly expressed in GC cell line than that in GES-1 (Fig. 1F-G).

### 3.2. Knockdown of TRIM28 inhibits the proliferation and clone formation of GC cells

**Figure 2:**
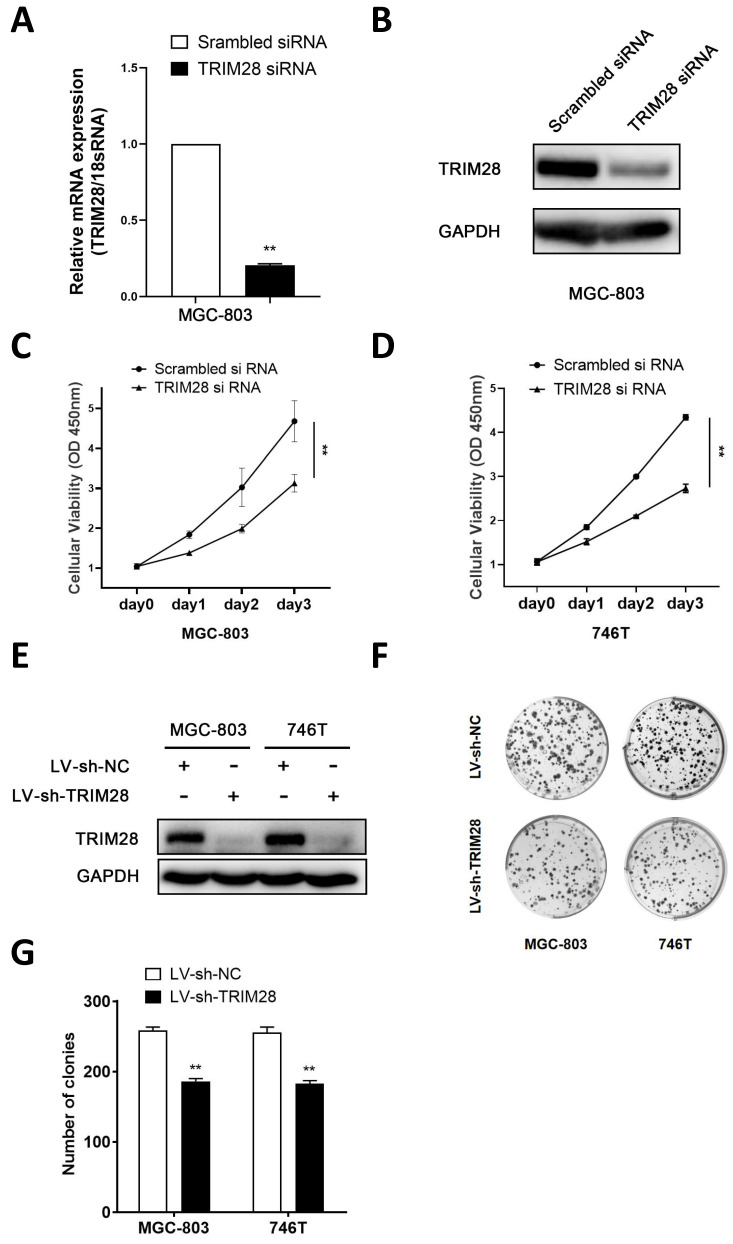
** Knockdown of TRIM28 inhibits the proliferation and clone formation of GC cells.** (A-B) qPCR and WB was performed to validate the transfection efficiency of TRIM28 siRNA into MGC803 for 48h. (C-D) CCK-8 assay was used to detect the proliferation of GC cells after TRIM28 knockdown. (E) TRIM28 knockdown lentivirus infected MGC803 and 746T cells were selected with puromycin for 2 weeks and validated by WB. (F) Representative images showing the results of colony formation assays after TRIM28 knockdown lentivirus transfection. (G) Statistical analysis of colony formation assay results. All the experimental results are shown as the mean ± SE of at least three independent biological replicates (n = 3) for each group. *P < 0.05; **P < 0.01.

To further explore the function of TRIM28 in GC cells, a TRIM28 knockdown model was established via the transfection of small interfering RNA and lentivirus. The qPCR and WB analyses revealed a notable reduction in the expression of TRIM28 in TRIM28-knockdown MGC803 cells (Fig. 2A-B). The CCK-8 assay revealed that TRIM28 knockdown significantly inhibited the proliferation of MGC-803 and 746T cells compared with the controls (*P* < 0.05, Fig. 2C-D). The WB results showed that the protein expression of TRIM28 was significantly downregulated after TRIM28-knockdown lentivirus infection into MGC803 and 746T cells (Fig. 2E). The colony formation assays demonstrated a significant reduction in the number of clones in the TRIM28-knockdown group (*P* < 0.05, Fig. 2F-G).

### 3.3. TRIM28 regulates IDO1 expression in GC

**Figure 3:**
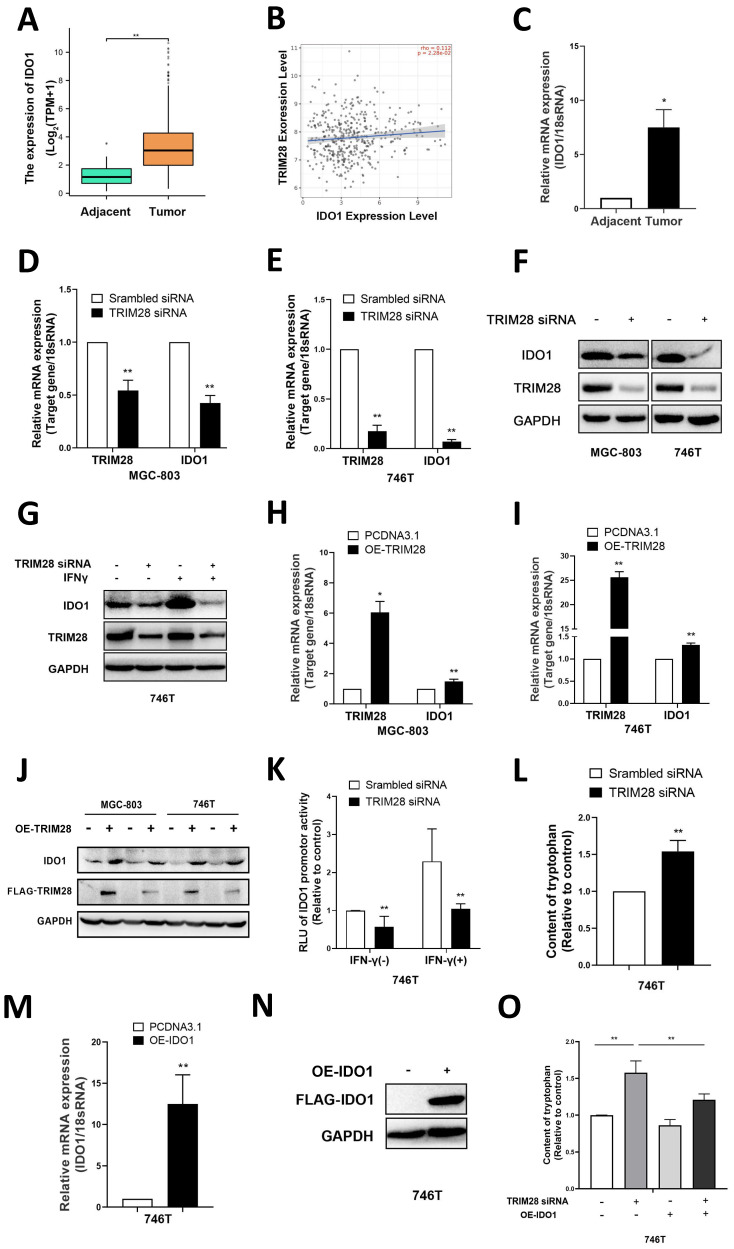
** TRIM28 regulates IDO1 expression in GC.** (A) Comparison of IDO1 mRNA expression between GC tissues and adjacent tissues in the TCGA database. (B) Scatter plot showing a positive correlation between the mRNA expression of IDO1 and TRIM28 in gastric cancer. (C) mRNA expression of IDO1 in GC tissues and adjacent tissues was measured by qPCR. (D-E) Verification of TRIM28 and IDO1 mRNA expression in MGC803 and 746T cells after TRIM28 knockdown for 48h by qPCR. (F) Representative images of WB showing the protein expression of TRIM28 and IDO1 after TRIM28 siRNA transfection into MGC803 and 746T cells for 48h. (G) After treated with TRIM28 siRNA for 48h and IFN-γ for 24h, protein levels of IDO1 were measured by WB. (H-I) mRNA expressions of TRIM28 and IDO1 in MGC803 and 746T cells after overexpression of TRIM28 for 48h were measured by qPCR. (J) Protein expressions of TRIM28 and IDO1 after TRIM28 plasmid transfection into MGC803 and 746T cells for 48h by WB. (K) Dual luciferase assay showed the activity of the IDO1 promoter after TRIM28 knockdown. (L) Fluorometric assay was used to measure cellular supernatant tryptophan levels in TRIM28-knockdown MGC803. (M-N) qPCR and WB were conducted to validate the transfection efficiency of the IDO1 plasmid in 746T cells. (O) Tryptophan Fluorometric Assay Kit was performed to detect the tryptophan content in the supernatants. All the experimental results are shown as the mean ± SE of at least three independent biological replicates (n = 3) for each group. *P < 0.05; **P < 0.01.

To further elucidate the possible downstream genes that are regulated by TRIM28 in GC, RNA sequencing was conducted to explore differentially expressed genes (DEGs) between the TRIM28-knockdown group and control groups. The results showed that IDO1 expression was significantly downregulated (data not shown). A series of human cancers over-express IDO1 in a constitutive way 26,27. In accordance with this, our bioinformatics analysis of the TCGA-STAD cohort revealed a significant increase in the mRNA expression of IDO1 in GC tissues (*P* < 0.01, Fig. 3A). And according to the results of the TIMER 2.0 database, there was a positive correlation between IDO1 and TRIM28 expression in GC (*P* < 0.05, r=0.112, Fig. 3B). Subsequently, qPCR was performed to measure the expression of IDO1 in gastric tissues from clinical patients, and the results indicated that the mRNA of IDO1 was also significantly upregulated in GC (*P* < 0.01, Fig. 3C).

Subsequently, qPCR was conducted to validate the DEGs. TRIM28 knockdown significantly downregulated the mRNA and protein expression of IDO1 in GC cells (*P* < 0.01, Fig. 3D-F) and reversed the IFN-γ-induced upregulation of IDO1 (Fig. 3G).

In contrast, overexpression of TRIM28 upregulated the mRNA and protein expression of IDO1 in MGC-803 and 746T cells (*P* < 0.05, Fig. 3H-J). Moreover, the luciferase assay results showed that TRIM28 siRNA inhibited the activity of the IDO1 promoter in 746T cells and reversed the IFN-γ-induced IDO1 promoter activity (*P* < 0.01, Fig. 3K). It suggests that TRIM28 may regulate IDO1 expression levels through the transcription factor pathway. Previous studies have shown that IDO1 can catalyze the metabolism of tryptophan to kynurenine, inhibit the proliferation of CD8^+^ T cells, promote the accumulation of Treg cells, and thereby suppress specific immunity 28. Therefore, a fluorometric assay was used to measure cellular supernatant tryptophan levels, and knockdown of TRIM28 resulted in a significant increase in the levels of tryptophan in cell supernatants (*P*<0.01, Fig. 3L). And the elevated tryptophan level caused by TRIM28 knockdown could be reversed by IDO1 overexpression (*P*<0.01, Fig. 3M-O). These results indicated that TRIM28 may regulate the expression of IDO1 to influence its function of metabolism of tryptophan. All of the above results indicate that TRIM28 may exerts its biological function in gastric cancer by regulating IDO1.

### 3.4. TRIM28 regulates IDO1 expression dependent on SRF

**Figure 4:**
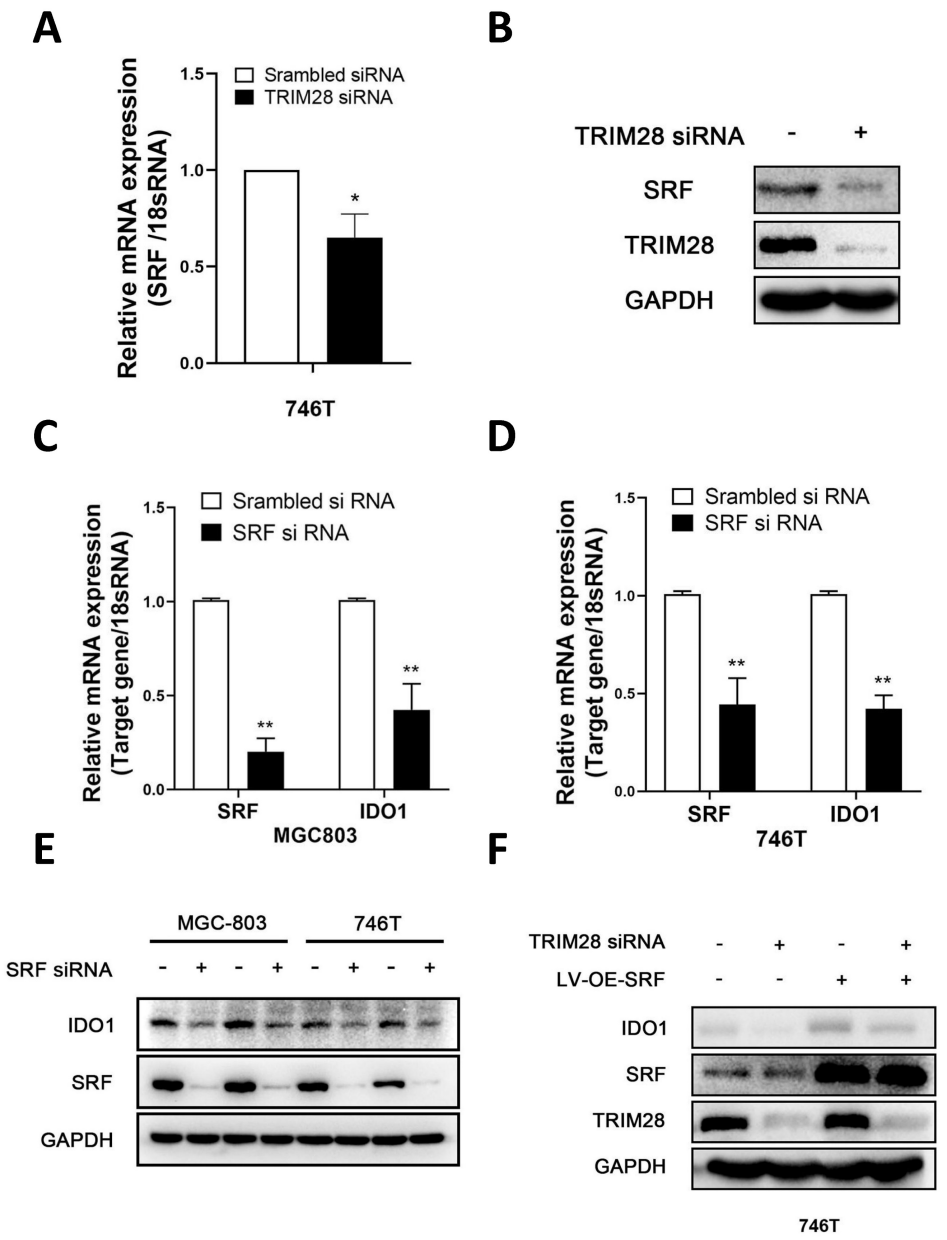
** TRIM28 regulates IDO1 expression dependent on SRF.** (A-B) qPCR and WB were performed to detect the expression of SRF after TRIM28 knockdown for 48h. (C-E) Detection of IDO1 mRNA and protein expression in 746T and MGC803 cells after SRF knockdown by qPCR and WB. (F) 746T cells stably expressing LV-OE-SRF/LV-NC was transfected with TRIM28 siRNA/scrambled siRNA for 48h and WB was conducted to detect the expression of IDO1 protein. All the experimental results are shown as the mean ± SE of at least three independent biological replicates (n = 3) for each group. *P < 0.05; **P < 0.01.

Considering that IDO1 is a downstream gene of TRIM28, we further investigated the specific mechanisms by which TRIM28 regulates IDO1. SRF has been reported as a classical transcription factor of IDO1 29. In our results, SRF mRNA and protein levels were found to be decreased after knockdown of TRIM28 in 746T cells (*P* < 0.05, Fig. 4A-B). Decreased mRNA and protein expression levels of IDO1 were detected after SRF knockdown in 746T and MGC803 cells (*P* < 0.05, Fig. 4C-E). Furthermore western blot showed that SRF overexpression could reverse the decrease of IDO1 protein expression level caused by TRIM28 knockdown in 746T cells (*P* < 0.05, Fig. 4F). These results suggest that TRIM28 may regulate IDO1 expression through SRF.

### 3.5. TRIM28 regulate GC cell proliferation through IDO1

**Figure 5:**
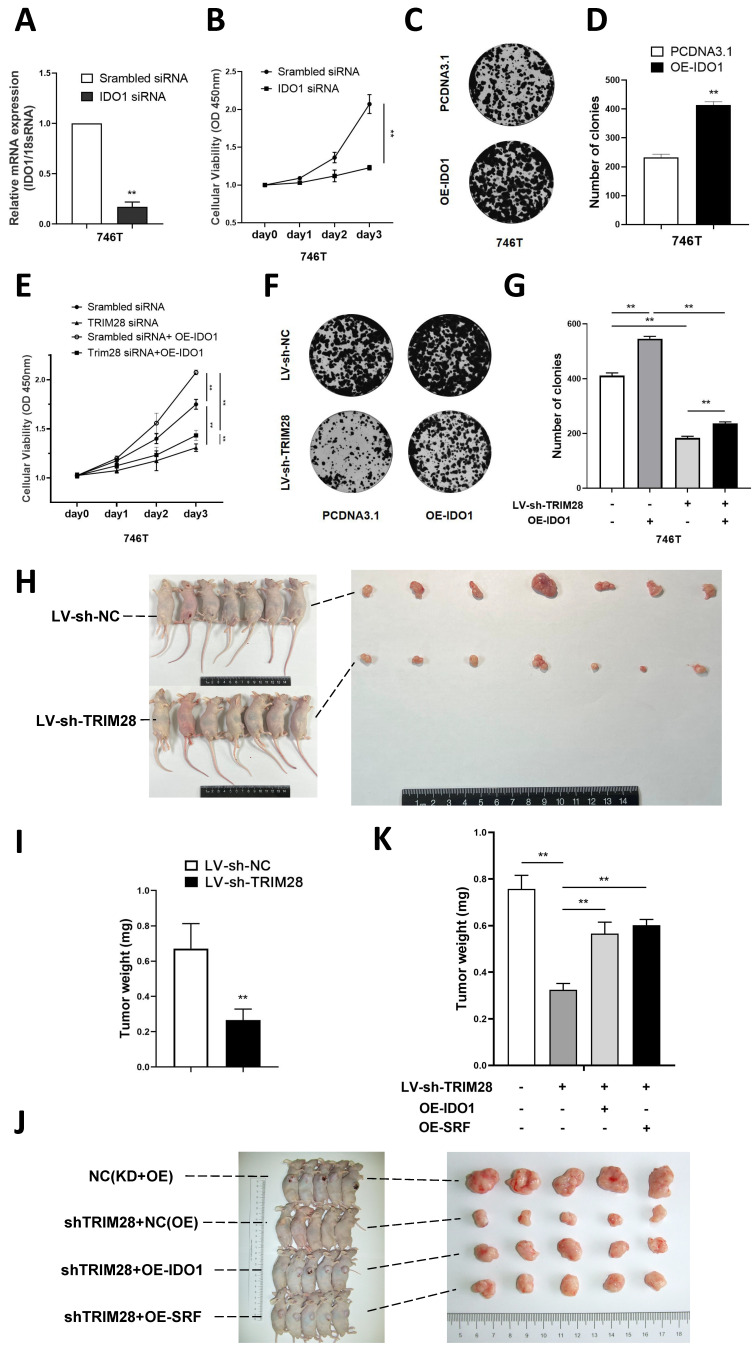
** TRIM28 regulate GC cell proliferation through IDO1** (A) Histogram plot showing confirmation of the transfection efficiency of IDO1 siRNA in 746T cells by qPCR. (B) CCK-8 assay was used to detect the proliferation of GC cells after IDO1 knockdown. (C) Representative images showing the results of colony formation assays after IDO1 plasmid transfection. (D) Statistical analysis of colony formation assay results. (E) CCK-8 assay was used to detect the proliferation of GC cells after TRIM28 knockdown lentivirus and overexpressed IDO1 plasmid transfection. (F) Representative images showing the results of colony formation assays after TRIM28 knockdown lentivirus and overexpressed IDO1 plasmid transfection. (G) Statistical analysis of colony formation assay results. (H-I) Representative images of subcutaneously transplanted tumors with MGC-803 cells in nude mice and statistical analysis of tumor weight. (J-K) Representative images of subcutaneously transplanted tumors with 746T cells in nude mice and statistical analysis of tumor weight. All the experimental results are shown as the mean ± SE of at least three independent biological replicates (n = 3) for each group. *P < 0.05; **P < 0.01.

The CCK-8 assay results showed that knocking down IDO1 significantly inhibited the proliferation of 746T cells (*P* < 0.01, Fig. 5A-B). Meanwhile, IDO1 overexpression could significantly increase the number of clones of 746T cells (*P* < 0.05, Fig. 5C-D). To further investigate whether TRIM28 regulates cell proliferation through IDO1, we transfected an IDO1 overexpression plasmid into GC cells and found that the IDO1 overexpression plasmid could partly reverse the effect of TRIM28 knockdown on inhibiting cell proliferation by CCK8 and clone formation assays (*P* > 0.05, Fig. 5E-G). These results indicate that TRIM28 may regulate cell proliferation through IDO1 in GC.

To investigate the effect of TRIM28 *in vivo*, we first established a TRIM28 knockdown model in MGC803 cells through lentivirus transfection. Subsequently, nude mice were injected with either TRIM28 knockdown MGC803 cells or control cells to establish a subcutaneous xenograft tumor model. The growth of subcutaneous xenografts derived from mice injected with TRIM28-knockdown MGC803 cells exhibited a significant inhibition (*P* < 0.01, Fig. 5H-I). In order to investigate whether TRIM28 regulates gastric cancer cell proliferation through the SRF/IDO1 axis *in vivo* experiments, we performed recovery experiments. We transfected TRIM28 knockdown lentivirus combined with overexpressed IDO1 plasmid or overexpressed SRF lentivirus into 746T cells. By constructing a subcutaneous xenografts model in nude mice, we found that both SRF and IDO1 overexpression could partly reverse the diminished proliferative ability of gastric cancer cells caused by TRIM28 knockdown (*P* < 0.01, Fig. 5J-K).

## Discussion

This study preliminarily explored the function and molecular mechanism of TRIM28 in regulating the proliferation of GC cells. We identified TRIM28 as a novel regulator of GC development as shown by the impaired proliferation in TRIM28 knockdown GC cells and subcutaneous xenograft. We demonstrated that TRIM28 ablation decreased the expressions of IDO1 and SRF. We identified that SRF mediated the effects of TRIM28 on expressions of IDO1 and IDO1 mediated the effects of TRIM28 on GC proliferation. Taken together, our results strongly indicate that TRIM28 is required for SRF/IDO1 signaling pathway during GC proliferation.

The occurrence of tumors is regulated by a combination of multiple factors, ultimately leading to uncontrolled cell proliferation and tumor progression. Previous studies have shown that TRIM28 is involved in the regulation of many biological processes, such as cell development and proliferation, as well as the occurrence and development of tumors. The role of TRIM28 in gastric cancer cells is still controversial. Zou C reported that BHRF1 triggered the expression of SNHG8, which sponged miR-512-5p and upregulated TRIM28 and a set of effectors to promote EBVaGC tumorigenesis and invasion 30. Ma X found that TRIM28 promotes the escape of gastric cancer cells from immune surveillance by increasing PD-L1 abundance 11.

The analysis of the data from a cohort of patients with GC revealed that increased levels of TRIM28 were associated with a decreased overall survival rate 31. These researches indicate that TRIM28 promotes GC development. However, it was also showed that although TRIM28 was highly expressed in GC tissues than peritumoral tissues, high expression level of TRIM28 in GC was associated with good prognostic effects 12. Moreover, TRIM28 knockdown enhanced the proliferation and clone formation of GC cell and enhanced the expression level of stemness markers through Wnt/β-catenin signaling pathways 12. Then in our study, we also observed TRIM28 was abnormally high protein and mRNA expression of TRIM28 both in GC tissues and cells, but was associated with bad prognostic effects. Cellular functional and *in vivo* experiments have shown that TRIM28 knockdown can inhibit the proliferation of GC cells, suggesting its potential involvement in the onset and progression of GC. And its specific molecular mechanisms deserve to be validated.

TRIM28 can also regulate downstream molecules, such as E2F1, p53, p73, AMPK-α1 and NF-κB, by acting as an E3 ubiquitination ligase or through its small ubiquitin-related modifier activity, and then affect tumor-related gene expression, EMT, or autophagy 32,33. Our results showed, that TRIM28 knockdown could inhibit the expression of IDO1. As previously mentioned, IDO1 is highly expressed in various tumor tissues and can regulate tumor immune responses. We also observed an abnormal increased IDO1 expression in GC tissues, which was positively correlated with the expression of TRIM28. Recent studies have found that the metabolite of IDO1, kynurenine, is transported into cells via SLC7A11 and inhibits ferroptosis signaling in tumors 34, suggesting that IDO1 can promote tumor development in a manner that is independent on immunosuppression. In fact, we observed that IDO1 exhibited positive correlation with GC proliferation. Furthermore, overexpression of IDO1 could partially reverse the inhibited effect of TRIM28 knockdown on GC cell proliferation. These results preliminarily suggest that TRIM28 may regulate the proliferation of GC cells through IDO1.

We have innovatively discovered the relationship between TRIM28 and IDO1 for the first time, but the specific mechanism by which TRIM28 regulates IDO1 is still unclear. A promoter activity assay revealed that TRIM28 knockdown can inhibit the promoter activity of IDO1, indicating that TRIM28 may regulate the mRNA expression of IDO1 at the transcription level. SRF is a member of the MADS BOX superfamily of transcription factors, which can regulate the expression of many genes by binding to SRF response elements in gene promoters and thus participate in what activities such as cell proliferation, differentiation, apoptosis, and cyclic regulation 35. SRF has been demonstrated to be associated with cancer cell progression and metastasis in a wide variety of tumors such as gastric and colorectal cancers 36,37. Direct SRF binding to the IDO1 gene promoter upregulated transcription of IDO1 was observed in oral squamous cell carcinoma (OSCC). Inhibiting IDO1 impaired SRF-induced migration and invasion and prevented epithelial-to-mesenchymal transition (EMT) in OSCC cells 29. We found that TRIM28 was positively correlated with the expression of SRF in GC cells. Our results also showed that decreased SRF inhibited the expression of IDO1 in GC cells. Moreover, Overexpressed SRF was able to partly reverse the reduced IDO1 expression by TRIM28 knockdown, indicating that TRIM28 may regulates IDO1 expression through SRF in GC. And specific mechanisms of SRF regulation by TRIM28 is also one of our directions for future in-depth research. Furthermore, the suppressed effect of TRIM28 knockdown on GC cell proliferation could be partially restored by overexpressing SRF *in vivo* experiment. These results demonstrate that TRIM28 may regulate the proliferation of GC cells through SRF.

We revealed a novel molecular mechanism SRF/IDO1 through which TRIM28 modulates GC proliferation. This should provide potential targets or biomarkers for GC diagnosis and treatment. The results of recent clinical trials on IDO1-targeting drugs are not satisfactory, indicating that the effect of IDO1 may be influenced by other factors. Our experimental results also provide some new insights for the targeting of IDO1 in clinical treatment, such as whether measuring both IDO1 and TRIM28 levels can more accurately identify the population of patients for whom IDO1-targeting drugs are most suitable. Of course, our article has certain limitations. For example, further research is needed on the key target genes and signaling pathways by which TRIM28/IDO1 regulates cell proliferation, including the specific molecular mechanisms by which TRIM28 regulates SRF. Whether TRIM28 expression level assay is effective in assessing the therapeutic efficacy of IDO1-targeted drugs is also one of the directions of our future research.

## Conclusions

Our study suggested that TRIM28 is crucial in the development of GC, and may regulate the proliferation of GC cells through SRF/IDO1 axis.

## Figures and Tables

**Table 1:** Univariate analysis of TRIM28 IHC and clinical variables.

Clinicopathologic parameters	TRIM28 protein		*P*
	-	+	
	(n=23)	(n=53)	
**Age(years)**			
≤60	8	20	0.806
>60	15	33	
**Gender**			
Male	11	32	0.311
Female	12	21	
**Tumor size (cm)**			
≤5	14	18	0.029
>5	9	35	
**Lauren classification**			
Intestinal	7	25	0.175
Diffuse	16	28	
**Differentiation**			
Well, moderately	12	21	0.311
Poorly, undifferentiated	11	32	
**Local invasion**			
T1, T2	5	14	0.665
T3, T4	18	39	
**Lymph node metastasis**			
No	4	10	0.879
Yes	19	43	
**TNM stage**			
I, II	10	16	0.262
III, IV	13	37	

**Table A1:** The primer sequences of genes

Primer name	Forward sequences (5'-3')	Reverse sequences (5'-3')
TRIM28	CGCCTTGGGGACAAACAT	CAGTCACCTTCTGGGCATCA
IDO1	GCGCTGTTGGAAATAGCTTC	CAGGACGTCAAAGCACTGAA
SRF	GCCACTGGCTTTGAAGAGAC	GGTGCCAGGTAGTTGGTGAT
18sRNA	GTAACCCGTTGAACCCCATT	CCATCCAATCGGTAGTAGCG
